# Adenosine stress perfusion CMR accurately identifies the culprit vessel

**DOI:** 10.1186/1532-429X-13-S1-P104

**Published:** 2011-02-02

**Authors:** James D Richardson, Angela G Bertaso, Dennis Wong, Adam J Nelson, Hussam Tayeb, Benjamin K Dundon, Payman Molaee, Kerry Williams, Matthew I Worthley, Karen S Teo, Stephen G Worthley

**Affiliations:** 1 Cardiovascular Research Centre, Royal Adelaide Hospital & Department of Medicine, University of Adelaide, Adelaide, Australia; 2 Cardiovascular Research Centre, Royal Adelaide Hospital, Adelaide, Australia

## Objective

To determine the ability of adenosine stress perfusion cardiac magnetic resonance (CMR) to accurately identify the culprit vessel.

## Background

The diagnostic evaluation of patients with suspected ischaemic heart disease (IHD) frequently involves a functional assessment of ischaemia. Adenosine stress perfusion CMR is a non-invasive test with high sensitivity for the detection of IHD, however rarely is its accuracy for culprit vessel identification assessed. We sought to determine the accuracy of stress perfusion CMR for identifying the culprit vessel. Furthermore, we sought to affirm the specificity of a positive CMR against an angiographic gold standard.

## Methods

Retrospective study of patients with a positive stress perfusion CMR and subsequent coronary angiography. Perfusion imaging was obtained at stress (adenosine 140 µg/kg/min) and rest on a 1.5T scanner. Late enhancement was assessed with dual pass gadolinium (0.2mmol/kg total dose). Angiographic stenosis ≥ 50% was defined as significant. The presence or absence of a significant lesion together with the correlation between ischaemic territory on CMR and angiographic culprit vessel was evaluated.

## Results

Thirty seven patients (60% male, age 65.1 years ± 11.3; mean ± SD) had a positive CMR with subsequent angiography, with follow up data for a median 24 months (IQR 21-27 months). Thirteen patients (35%) had previous myocardial infarction or revascularisation. Of the cohort of 37, six (16%) had normal angiograms and 31 (84%) had a significant epicardial stenosis. Of the six false positives, three had localised septal hypoperfusion, while a further three had circumferential defects. Of the 31 patients correctly identified, CMR accurately established the territory of the culprit vessel in 29 (94%). The vessels confirmed as ischaemic were left anterior descending 12 (39%), circumflex 6 (19%) and right coronary artery 13 (42%).

## Conclusions

Adenosine stress perfusion CMR reliably identifies the territory supplied by the culprit vessel (29 out of 31 - 94%). Furthermore, we reaffirm the high specificity (84%) of stress perfusion CMR.

